# Y-chromosomal genes affecting male fertility: A review

**DOI:** 10.14202/vetworld.2016.783-791

**Published:** 2016-07-30

**Authors:** Jasdeep Kaur Dhanoa, Chandra Sekhar Mukhopadhyay, Jaspreet Singh Arora

**Affiliations:** School of Animal Biotechnology, Guru Angad Dev Veterinary and Animal Sciences University, Ludhiana - 141 004, Punjab, India

**Keywords:** holandric genes, male fertility, microdeletion sex-chromosome, spermatogenesis

## Abstract

The mammalian sex-chromosomes (X and Y) have evolved from autosomes and are involved in sex determination and reproductive traits. The Y-chromosome is the smallest chromosome that consists of 2-3% of the haploid genome and may contain between 70 and 200 genes. The Y-chromosome plays major role in male fertility and is suitable to study the evolutionary relics, speciation, and male infertility and/or subfertility due to its unique features such as long non-recombining region, abundance of repetitive sequences, and holandric inheritance pattern. During evolution, many holandric genes were deleted. The current review discusses the mammalian holandric genes and their functions. The commonly encountered infertility and/or subfertility problems due to point or gross mutation (deletion) of the Y-chromosomal genes have also been discussed. For example, loss or microdeletion of sex-determining region, Y-linked gene results in XY males that exhibit female characteristics, deletion of RNA binding motif, Y-encoded in azoospermic factor b region results in the arrest of spermatogenesis at meiosis. The holandric genes have been covered for associating the mutations with male factor infertility.

## Introduction

The sex-chromosomes are critical players in determining the sex of almost all of the multicellular organisms. The Y-chromosome is one of the two sex-chromosomes in mammals and is usually the smallest chromosome in the karyotype. Y-chromosome is suitable for studying the evolutionary relics, speciation, and male infertility and/or subfertility due to its unique features such as long non-recombining region and holandric inheritance pattern [1]. Nettie Maria Stevens (along with Edmund Beecher Wilson) identified that Y-chromosome is a sex-determining chromosome as early as in 1905 while conducting one study on the mealworm (*Tenebrio molitor*) [2]. She also proposed that chromosomes always existed in pairs. All chromosomes normally appear to assume a well-defined shape during mitosis when observed under a microscope. This shape is vaguely X-shaped for all chromosomes. Interestingly, the Y-chromosome looks like the English alphabet “Y” during mitosis due to merging of the two very short branches [3]. The X- and Y-chromosomes are thought to have evolved from a pair of identical chromosomes, known as autosome. The mammalian males are heterogametic (produce X- and Y-chromosomes bearing sperms in equal proportion), and the females are homogametic (all ova are X-bearing). It was found that Y-chromosome has high repetitive DNA sequence content, which consists of pseudogenes and does not have any function [4].

## Structure and Organization of Y-chromosome

Y-chromosome is the smallest chromosome that consists of 2-3% of the haploid genome and may contain between 70 and 200 genes [5]. In general, the mammalian Y-chromosome has two arms that harbor two pseudoautosomal regions (PAR1 and PAR2), which recombine with their homologous regions on X-chromosome; and a male-specific Y region (MSY) (or non-recombining region on Y [NRY]), which does not recombine with the X-chromosome. The absence of recombination makes the genetic mapping of the Y-specific region impossible, and the complexity of the repetitive sequences makes the physical mapping of the Y-specific region difficult. These PAR1 and PAR2 consist of 5% of the entire chromosome [5]. Being male-specific, the holandric genes are mostly involved in male sex determination, fertility, and development. Y-chromosome is present in different shapes and sizes in different species. In humplesscattle (*Bostaurus*), it is a small submetacentric, whereas in humped zebu (*Bosindicus*), it is a small acrocentric with visible small p-arm (“p” for “petite,” means small); in river buffalo (*Bubalusbubalis*), it is a small acrocentric chromosome, whereas in both sheep (*Ovisaries*) and goat (*Capra hircus*), it is very small and metacentric [6,7]. The repeat sequences of Y-chromosomes have also been studied to associate with spermatological parameters [8].

## Important Genes Harbored by Mammalian Y-chromosomes

In 1976, Tiepolo and Zuffardi first reported the involvement of Y-chromosome in male infertility [9]. It was hypothesized that during the course of evolution the Y-chromosome has acquired a large number of testes-specific genes responsible for spermatogenesis and any deletion in these genes results in infertility [10]. Mukhopadhyay reviewed that sequencing of human Y-chromosome has revealed that there are 34 genes in human PAR and 156 known transcription units in human MSY [11]. The microdeletions at Yq11 represent etiological factor with idiopathic azoospermia or oligozoospermia [11]. The SRY gene (sex determining region of Y) is responsible to elicit testis determination and male development. Loss or microdeletion of SRY gene results in XY male exhibiting feminine characteristics (known as Swyer syndrome or XY gonadal dysgenesis) [12]. Some of the important genes, *viz*., regions (azoospermic factor [AZF]) on the mammalian Y-chromosome have been discovered and later characterized. These genes have been covered in this review. Important features such as cytogenetic location, function, and mutational deficiencies associated with some of the holandric genes are adumbrated in the Table-1.

**Table-1:** Cytogenetic location, major functions, salient features, and disorders associated with aberrations associated with mammalian Y-chromosome.

Gene name, size, location*	Expression	Role/Function	Disorder
USP9Y, 2555 aa, Yq11.2	Embryonic and adult tissues including testis	Spermatogenesis	Sertoli cell only syndrome, Y-chromosome based infertility, impaired or no sperm production [69]
BPY2, 106 aa, Yq11.223	Testis	Male germ cell development, male infertility	Y-chromosome infertility, male infertility, spermatogenic failure, Y-linked 2 [70]
RBMY, 391 aa	Developing germ cells	Transcriptional regulation processes, tumor suppression	Infertility in males [17], deletion in mice results in high level of sperm development [16]
DAZ, 744 aa, Yq11.223	Restricted to pre-meiotic germ cells, particularly in spermatogonia	Spermatogenesis, translational regulation, promotes germ-cell progression to meiosis and formation of haploid germ cells	Persistent fetal circulation syndrome, azoospermia, deletion of DAZL1 in mouse results in complete absence of gamete production and reduced number of germ cells [18]
SRY, 204 aa, Yp11.3	Adult testis by germ cells	Sex determination acts as transcriptional regulator, promotes DNA bending, involved in premRNA splicing	Mixed gonadal dysgenesis, 46XYsex reversal 1, XX male syndrome [21]
TSPY, 308 aa, Yp11.2	Testis, tumor germ cells of gonadoblastoma specimens	Sperm differentiation and proliferation, cell cycle regulation, transcription regulation, neurotransmission, meiotic division, tumor suppression and promotion	Gonadoblastoma, sudden infant death with dysgenesis of the testis syndrome, infertility, cancers, diabetes, and neural dysfunctions [71]
AmelogeninAMELY-206 aa, Yp11.2 [29]	Testis, ovary, lungs, liver (based on microarray)	Organizing of enamel rods during tooth development, biomineralization	Dental pulp necrosis, dental fluorosis, amelogenesisimperfecta [29], deleted amelogenin males [31]
ZFY, 801aa, Yp11.3	Testis	Transcriptional activator, sperm or testis maturation	Cystadenofibroma, campomelic dysplasia [32]
DDX3Y, 660 aa, Yq11.2	Testis, placenta, ovary, uterus, lung, liver, kidney (based on microarray)	Translation initiation, nuclear and mitochondrial splicing, embryogenesis	Spermatogenesis, male infertility, sertoli cell only syndrome, severe hypospermatogenesis [36]
HSFY, 401 aa, Yq11.22	Testis, sertoli cell, spermatogenic cells	Transcriptional activators	Male infertility, azoospermia, sertoli cell only syndrome, oligospermia [72]
KDM5D, 1539 aa, Yq11	Testis, ovary, whole blood, kidney (based on microarray)	Histone coding	Y-chromosome infertility[14]
RPS4Y2, 263 aa, Yq11.22	Testis, prostate, whole blood (based on RNAseq)	Spermatogenesis	Y-chromosome infertility [39]
PRY, 147 aa, Yq11.22	Testis	Spermatogenesis, apoptosis of defective spermatozoa	Y-chromosome infertility, azoospermia
CDY, 598 aa, Yq11.23	Testis	Chromatin targeting and maximal enzymatic activity of PRC2, acts as a positive regulator of PRC2 activity	Gingival recession
AKAP17A, 695 aa, Yp11.32	Testis, prostate, ovary (based on microarray and RNAseq)	Regulation of pre-mRNA splicing	Chronic tic disorder
CD99, 185 aa, Yp11.3	Testis, prostate, ovary (based on microarray and RNAseq)	T-cell adhesion and spontaneous rosette formation with erythrocytes, helping leukocytes to overcome the endothelial basement membrane	Testicular fibroma, testicular granulosa cell tumor
TGIF2LY, 185 aa, Yp11.2	Testis (based on RNAseq)	Transcription role in testis, act as a competitor/regulator of TGIF2LX	Prostate cancer [49]
TBL1Y, 522 aa, Yp11.2	Testis, prostate, ovary (based on microarray and RNAseq)	Transcription activator	Deafness sensorineural [73]
VCY, 125 aa, Yq11.22	Testis, male germ cells	Spermatogenesis Sex ratio distortion	Y-chromosome infertility, X-linked ichthyosis, gonadoblastoma [53]
NLGN4Y, 816 aa, Yq11.22	Testis, prostate, ovary (based on microarray and RNAseq)	Cell-cell interactions	Asperger syndrome, tuberculosis, autistic disorder [54]

* Cytogenetic location in human. PRC2=Polycomb repressive complex 2

## Functions of Genes on Y-chromosome

### AZF

AZF, distributed through three genetic regions (namely, AZFa, AZFb, and AZFc), is name of a region (not a single gene) on the long arm of human Y-chromosome (Yq11.2: “q” for queue means tail) that shelters a number of protein coding genes associated with spermatogenesis [5]. The AZFa harbors some single copy genes (involved in spermatogenesis) that have X-homologs. The overlapping AZFb and AZFc regions are jointly termed as AZF2. In AZFb region, the eukaryotic translation initiation factor 1A, Y-linked (*EIF1AY*) and RNA binding motif, and Y-encoded (*RBMY*) genes have been mapped in man [13]. EIF1A protein is involved in stabilizing the binding of the initiator methionine-bearing-tRNA to 40S ribosomal subunits. The AZFc region harbors five genes: Deleted in azoospermia (*DAZ*), chromodomain Y1, basic protein Y2 (*BPY2*), PTA-BL related Y (*PRY*), and testis transcript Y2 [14].

### Ubiquitin-specific peptidase 9, Y-linked (*USP9Y*)

USP9Y (initially known as *Drosophila* fat facets related Y) is a single copy gene, located in the AZFa region. USP9Y enzyme belongs to the peptidase C19 family and has role in sperm cell development in mammals. USP9Y enzyme is similar to the ubiquitin-specific proteases, which cleave the ubiquitin moiety from ubiquitin-fused precursors and ubiquitinated proteins [14].

### BPY2

This male-specific gene encodes testis-specific *BPY2* in mammals, which interacts with ubiquitin protein ligase E3A, which are enzymes that target other proteins to be broken down (degraded) within cells. These enzymes attach a small protein called ubiquitin to proteins that should be degraded. The gene is located in two copies within a palindromic region at Hsa-Yq11 in man.

### Protein kinase, Y-linked pseudogene (*PRKY*)

PRKY is a serine/threonine-protein kinase enzyme that is encoded by the *PRKY* “transcribed pseudogene which is located on Y-chromosome near the PAR in human.” Hamamah *et al*. [15] isolated and characterized the *PRKY* gene in man, which is highly homologous to the *PRKX* gene on Xp 22.3. Sex reversal in human XX male and XY females results due to sex-chromosomal translocation (X;Y) (p22;p11) between proximal part of PRKY and its X-homologue PRKX [15].

### RBMY

RBMY encodes a germ cell-specific nuclear protein involved in spermatogenesis. It is a multicopy gene family with 30-40 members (nested in the AZFb region), most of which are pseudogenes. The encoded protein contains an RNA-binding motif in the N-terminus and four serine, arginine, glycine, and tyrosine (SRGY) boxes in the C-terminus. In men, six highly similar copies (RBMY1 to 6) have full-length ORFs and are considered functional [16]. RBMY shares high homology with an autosomal heterogeneous nuclear ribonucleoprotein G (*hnRNP-G*) gene that contains an RNA-binding motif and one of the four SRGY repeats found in RBMY, suggesting that RBMY functions as an RNA processing factor. The *hnRNP*-G is a nuclear protein whose function is unknown that binds nascent pre-mRNA *in vivo* and in nuclear extracts. RBMY1 siRNA(h) is recommended for the inhibition of RBMY1 expression in human cells [16]. *RBMX* is homologous of *RBMY* present on human Xp26. In mice, *RBMY* is not expressed in intermediate spermatocytes, but it is expressed discontinuously in the spermatogonia and elongated spermatids. RBMY gene is present in Y-chromosome of higher primates and is absent in other eutherian and marsupial species. Human and mouse *RBMY* have an X-chromosomal paralogue (*RBMX*), which encodes the widely expressed hnRNP-G [17].

### DAZ

The DAZ gene family, with three functional copies, *viz*., DAZ-1, -2, and -3, is expressed only in the mammalian male germ cells. Reports suggest that *DAZ* and DAZ-like 1 (*DAZL1*) genes control the cell cycle switch from mitotic to meiotic cell division through the RNA recognition motifs on these gene products [18]. The presence of a wide array of DAZ transcripts in single individual suggests that the transcripts could also have arisen by alternative splicing of a single gene. Four copies of this gene are found within the palindromic duplications on human Y-chromosome: One pair of genes as a part of the P1 palindrome and the second pair as a part of the P2 palindrome. Each gene consists of 2.4 kb repeat (that encodes 24 amino acids) with 72 bp exon [19]. The *DAZL1* is an autosomal gene present in all vertebrates while *DAZ* is found in old world monkeys. The cynDAZL1, isolated from the crab-eating cynomolgus monkey, contains one *DAZ* repeat and displays high homology to humans *DAZL1*. The immunolocalization studies of cynDAZL1/DAZL1 in cynomolgus monkey testis revealed a biphasic expression pattern with proteins being detectable in a range of developing sperm cells, *viz*., spermatogonia and late spermatocytes, however, not in early spermatocytes and late spermatids [18].

### SRY

*SRY*, are gulator of testis-determining factor (TDF), is an intronless, sex-determining gene that islocated on the short arm of Y-chromosome in man [20]. The most convincing evidence on the role of SRY on testis formation was obtained from the experiment of Wilhelm *et al*. [21], where they microinjected 14-kb region of DNA including the *SRY* gene (and its regulatory elements) into the genome of a normal XX mouse zygote [21]. The XX embryos were reported to develop testes, male accessory organs, and penis. The SRY-locus was the first identified as a member of the SOX transcription factor family [22]. SOX9 is an autosomal gene which is involved in sex determination. It encodes a transcriptional factor that contains high mobility group (HMG) box. *SRY* comprises of a conserved DNA binding domain (HMG-box) of a single exon, encoding 204 amino acid proteins which regulate gene expression. In fetal mice, *SRY* is expressed in somatic cells of genital ridge, while in sheep expression persists even after full differentiation of testis and in human and marsupials, *SRY* transcripts can also be detected in other fetal and adult tissues [23].

### Testes-specific protein, Y-encoded (*TSPY*)

This is a multicopy gene (between 20 and 40 among individuals), present in the NRY. This 33-kDa protein encoded by this gene is found only in testicular tissue and may be involved in spermatogenesis. The immune-histochemical data of *TSPY* expression suggested that it is responsible for the proliferation of germ cells [24]. Testes being the primary site of expression, the *TSPY* are expressed in spermatogonia, to lesser degree primary spermatocytes and in prostate-cancer tissue and cells. However, only a single, non-functional orthologous gene is found in mice. In human, two transcript variants encoding different isoforms have been found for this gene [25]. Gene isoforms are mRNAs that are produced from the same locus but are different in their transcription start sites. The gene is predominantly expressed in adult testes and to some extent in fetal testes due to the lower content of germ cell precursor stages [25]. *TSPY* is a good male-specific marker so can be used for sex determination in animals [26]. TSPY has been studied to identify SNPs associated with spermatological quality in cattle and buffalo [27, 28].

### Amelogenin

This extracellular matrix protein is involved in amelogenesis (i.e., development of enamel) of tooth. Each of the sex-chromosomes harbor one amelogenin gene. Sasaki and Shimokawa [29] first isolated and sequenced human *amelogenin* gene. This gene is present on both the sex-chromosomes in anthropoids (gorilla, chimpanzee, and orangutan) and bovines, some old world monkey (Japanese monkey, rhesus monkey, crab-eating macaque) but present on only X-chromosome in other old world monkey (baboon, patas monkey, and green monkey) [30]. Amelogenin gene is present in two forms, *viz*., *AmelX* and *AmelY*, which differ by a 6 bp deletion in *AmelX*. Polymerase chain reaction (PCR)-gel electrophoresis reveals two bands of DNA in males at 106 and 112 bp, and a single band at 106 bp in human females. It makes amelogenin a candidate of sex determination [31].

### Zinc finger gene, Y-chromosome (*ZFY*)

*ZFY* gene was originally an autosomal and was relocated to the X- and Y-chromosomes in eutherians along with neighboring genes, but it does not pass the marsupial test which indicates that it is not the ancestral TDF. In mice testicles, 2 zfy genes (*zfy1* and *zfy2*) are expressed while in human there is no report of function of zfy in testis [32]. The expression of these genes increases in germ cells entering meiosis. *ZFX* is the homologue of *ZFY* that encodes similar proteins and it is the regulator of self-renewal of hematopoietic stem cells and embryonic cells. Cloning and comparative analysis of *ZFX* and *ZFY* genes have been recently reported for bovine, porcine, and equine homologs. A high degree of similarity of bovine *ZFX* and *ZFY* genes was found to each other and with other species [33]. The *ZFY* gene is located on the R-band-positive region of Y-chromosome in cattle (exotic and zebu) and riverine buffalo [6].

### Ubiquitously transcribed tetratricopeptide repeat containing, Y-encoded (*UTY*)

In human, thesix mRNA transcripts of *UTY* gene translate into the enzyme histone demethylase UTY that contains tetra-trico-peptide repeats and are thought to be involved in protein-protein interactions. The UTY protein being a minor histocompatibility antigen could induce rejection of male stem cell grafts. Suppression of recombination between the homologous genes *UTY* and X-chromosomal *UTX*, results in a point mutation in mRNA transcripts of these genes, whereas the reading frames, *viz*., the protein domain structures are conserved [34]. UTY-derived peptides elicit immune-recognition in human and the mouse [35]. *UTY* plays major role in translational research on immunotherapy of leukemia, which prevent tumor relapse in post-transplant patients. In a male-recipient/female-donor setting, *UTY*-specific peptides exhibit a gender-specific antitumor effect, observed mainly *in vitro* [34].

### DEAD (Asp-Glu-Ala-Asp) box helicase 3, Y-linked or DEAD box protein 3, Y-linked (*DDX3Y*)

*DDX3Y* gene, also known as *DBY* gene, encodes a DEAD box protein which is characterized by the conserved motif Asp-Glu-Ala-Asp and is basically an enzyme [36]. It was reported that in mice treated with arsenic the expression of *DDX3Y* was down-regulated in testis and epididymis. This gene also has a homolog on X-chromosome (*DDX3X*) [36].

### Heat shock transcription factor, Y-linked (*HSFY1* and *HSFY2*)

HSFY belongs to HSF family and is involved in the spermatogenesis in animals as well as humans. *HSFY2*, a protein coding gene, is a candidate gene for azoospermia. This gene is present in two identical copies in genome with palindromic region. Realtime (RT)-PCR showed that *HSFY* family is largely expanded in cattle (?70 copies) as compared to human (2 functional copies, 4 HSFY-similar copies). *HSFY* expression in cattle appears to be restricted to the testis. The mRNA levels of *HSFY* varied significantly between bulls (p<0.0001) and correlates positively with mRNA markers of spermatogonial and spermatocytes [37].

### Lysine (K)-specific demethylase 5D (*KDM5D*) and ribosomal protein S4, Y-linked 2 (*RPS4Y2*)

KDM5D and RPS4Y2 both are protein coding encodes genes.*KDM5D* gene (also known as HY, HYA, SMCY, and JARID1D) encodes a protein containing zinc finger domains. A short peptide derived from this protein is a minor histocompatibility antigen which can lead to graft rejection of male donor cells in a female recipient. Alternative splicing results in multiple transcript variants [38]. RPS4Y2 is a protein of 40s ribosomal subunit, which is encoded by testis-specific RPS4Y2 gene in human [39].

### PTPN13-like, Y-linked (*PRY*)

*PRY*, a testis-specific protein-coding gene, was first reported by Lahn and Page [40]. This gene has four functional copies (PRY1-PRY4). The first two copies located in AZFb and encode a protein tyrosine phosphate that play role in spermatogenesis. *PRY3* and *PRY4* are located in AZFc region, and consist of 3, 4, or 5 exons. Yu *et al*. suggested that *PRY* has no significant role in spermatogenesis, but it is involved in apoptosis of mature sperm [41].

### Chromodomain protein Y-linked (*CDY*)

This gene consists of three members: One member located on Y-chromosome and two on autosomes (*CDYL* and *CDYL2*), which have been identified in human [42]. Two members *CDYL* and *CDYL2* mapped to human chromosome 6 and 16 and exist in most mammalian species [43]. The bCDLY and bCDLY2 genes harbor 9 and 7 exons, respectively, and these have been mapped to bovine chromosome 24 and 18, respectively.

### Protein A kinase or anchor protein 1 (*PKA* or *XE7*)

This pseudoautosomal gene in human is ubiquitously expressed. The structure and expression of this gene was identified in human tissues [44]. Alternative RNA splicing of XE7 results in two protein isoforms of 385 and 695 amino acids. The arginine/serine (R/S)-rich region in the larger of these suggests a role in mRNA processing [45]. With interaction of family of protein kinase A (PKA) anchoring proteins (AKAPs), PKA is targeted to distinct subcellular loci. Most AKAPs bind PKA through an amphipathic helix which consists of 14-18 amino acids that insert into a hydrophobic groove formed by the R-dimer as evident from resolution of nuclear magnetic resonance and crystal structures of the complex [46].

### *MIC2* gene for CD99 molecule

*MIC2* is another pseudoautosomal gene in man, which encodes a human cell surface molecule 12E7. MIC2 is a T-cell surface protein which is involved in aggregation of lymphocytes. Alternative splicing exhibits differential expression between the two forms, with the major form inducing cellular adhesion and the truncated form antagonizing this process (Santacruz Biotechnology, Inc.) [47]. Birch *et al*. reported the identification and characterization of *MIC* genes in human that were related to MHC Class I. They identified three *MIC* genes in the genome of cattle and located them close to three non-classical *MHC* Class I genes. Analyses of bovine cell line-specific MIC cDNA sequences indicate that in total, there may be four *MIC* genes [48].

### Transforming growth factor beta-induced factor homeobox 2-like, Y-linked (*TGIF2LY*)

This gene lies within the MSY region, in a block of sequence that is thought to be the result of a large X-to-Y transposition. The C-terminus of this protein is divergent from that of its X-chromosome homolog (*TGIF2LX*), suggesting that this protein may act as a regulator of *TGIF2LX*. This gene encodes a member of the TALE/TGIF homeobox family of transcription factors. Any change in *TGF2LY* gene results in azoospermia and infertility) [49]. TGIFLX/Y is homeodomain-containing genes with 2666-bp mRNA being encoded by two exons separated by a 96-bp intron. Comparative DNA analysis indicates that *TGIFLX* originated from retrotransposition of TGIF2, located on 20q11.2-12, onto the X-chromosome. RT-PCR analysis has revealed that X- and Y-linked genes were expressed in the adult testis [50].

### Transducin beta-like 1, Y-linked (*TBL1Y*)

The TBL1Y protein has sequence similarity with members of the WD40 repeat-containing protein family. Ramadoss *et al*. reported that TBL1 controls the expression of nuclear factor-κB (NF-κB) target gene (which controls tumor progression) by directly binding with NF-κB facilitating its recruitment to target gene promoters. TBL1 knockdown significantly reduced the invasive potential of breast cancer cells by inhibiting NF-κB [51]. TBL1Y is also involved in the genesis of non-syndromic coarctation of the aorta [52].

### Variable charge, Y-linked 1 (*VCY1*) and *VCY2*

These are variable charged proteins located in deleted AZFc region in infertile men. Members of the VCX/Y family share a high degree of sequence identity, with the exception that a 30-bp unit is tandemly repeated in X-linked members but occurs only once in Y-linked members [53]. *VCY2* is testis-specific gene and located in most frequently deleted AZFc region of Y-chromosome.*VCY2* is composed of 8 exons, of which, 5 are translated to amino acids. Cao *et al*. reported that due to the impaired expression of *VCY2* in infertile men, this gene is involved in the pathogenesis of male infertility [53].

### Neuroligin 4, Y-linked (*NLGN1*)

This gene encodes a Type I membrane protein that belongs to the family of neuroligins, which are cell adhesion molecules present at thepost-synaptic side of the synapse, and may be essential for the formation of functional synapses. An important paralog of this gene is *NLGN1*. Putative neuronal cell surface protein of this gene is involved in cell-cell interactions [54].

## Genetic Disorders Related to Y-chromosomal Anomalies

### Azoospermia

The reproductive disorder of male known as azoospermia is characterized by absence of sperm in ejaculate affecting ?20% of male fertility situations [55]. It can be classified as pre-testicular, testicular, post-testicular, and idiopathic azoospermia. A number of causes responsible for azoospermia includes: Primary or secondary testicular failure, Klinefelter syndrome [56], Y-chromosome microdeletions, genetic infertility due to abnormal chromosomes (karyotype) [41], Kallmann syndrome, hypothalamic/pituitary tumor, hyperprolactinemia, cancer treatment (chemotherapy, radiation, surgery), varicocele effect [57], pituitary suppression, diabetes mellitus, sickle cell anemia, hemochromatosis, sperm autoimmunity, undescended testicles at birth, obstruction, congenital absence of the vas deferens, ejaculatory duct obstruction, Young syndrome, and vasectomy [58]. Disease-like varicocele is associated with AZF1 (or AZFa) region of Y-chromosome [14]. EIF1A protein results in azoospermia when deleted. O’Brien *et al*. indicated that deletion of DAZ1/DAZ2 (however, not deletion of DAZ3/DAZ4) is associated with spermatogenic impairment [59]. The RBMY-deleted mice are fertile with normal sperm output [16]. However, RBMY knocked out mice exhibit normal spermatogenesis but develop structurally defective spermatozoa [17]. On the contrary, in human, deletion of *RBM* results in meiotic arrest and azoospermia. The diagnosis can be performed using karyotyping, PCR, or fluorescent *insitu* hybridization [60].

### Jacobs syndrome

It is a chromosomal genetic syndrome where the male person has an extra Y-chromosome, becoming XYY instead of normal XY (male) or XX (female). Immaturity, learning difficulties, arthritis, camptodactyly, swollen joints, and tall stature are the problems associated with Jacob’s syndrome [61].

### Klinefeltersyndrome (47,XXY karyotype)

This is an abnormality of sex-chromosome affecting 1 of 600 newborn males [62]. In this condition, an extra X-chromosome results in defective postnatal testicular function. This syndrome occurs due to non-disjunction of homologous chromosomes. During gametogenesis due to non-disjunctional event an extra chromosome has been retained by the gamete. Fertilization of a normal (X) egg with abnormal sperm having XY-chromosome produces an XXY offspring (Klinefelter) [63]. Hypogonadism has been suspected as a contributor to increase in morbidity and mortality due to Klinefelter syndrome [64]. This disorder is characterized by a 47, XXY or a mosaic karyotype, hypergonadotropic hypogonadism, infertility, reduced body hair and gynecomastia. The diagnosis can be performed by hormone testing or chromosomal analysis.

### Male infertility

Deletion of genetic material in the region of Y-chromosome (AZF) results in male infertility [65]. O’Brien *et al*. reported that the natural transmission of deletions which involves the *USP9Y* gene suggests that the absence of the *USP9Y* gene product does not preclude sperm-fertilizing ability, and thus, it is not critical for spermiogenesis [59]. This gene is present but inactive in chimpanzees and bonobos, however, it is absent (deleted) in infertile men [65]. Deletion in two of four holandric *DAZ* gene copies, which is difficult to detect due to multicopy nature, will result in spermatogenic failure [59]. HSFY gene has been mapped to the AZFb region of human Y-chromosome whose deletion results in male infertility [37]. Any change in *TGF2LY* gene results in azoospermia and infertility [66]. In Europe and the Western Pacific region, it is reported that partial *gr/gr deletion* of AZFc is associated withmale infertility among Caucasians [66]. Suganya *et al*. reported that male infertility may be caused by genetic abnormalities, varicocele, cryptorchidism, spermatic duct obstruction, urogenital tract infections, antisperm antibodies, endocrine disturbances, testicular malignancy, and environmental factors [67].

### Y-chromosome microdeletion

Missing genes in Y-chromosome result in microdeletions. Y-chromosome microdeletion is currently diagnosed by extracting DNA from leukocytes and mixing it with some of the about 300 known genetic markers for sequence-tagged sites on the Y-chromosome, and then using PCR amplification and gel electrophoresis to test whether the DNA sequence corresponding to the selected markers is present in the DNA [68].

## Conclusion

The advent of next generation sequencing and development of high end, faster bioinformatics tools and pipelines have enabled researchers to identify and annotate a number of novel genes. Until 1990s, the scientists speculated that the Y-chromosome is a barren land and it contains only SRY gene. However, at present at least a few dozens of Y-chromosomal genes have been studied. The Y-chromosomal genes have been explored associated with several male fertility-specific traits both in human and animals. In near future, some more new genes are expected to be explored and characterized using the most advanced sequencing technique, transcriptomics, proteomics, etc.

## Authors’ Contributions

All authors participated in the discussion, draf and revision of the manuscript. All authors read and approved the final manuscript.

## Competing Interests

The authors declare that they have no competing interests.
